# Angiographic correlations of patients with small vessel disease diagnosed by adenosine-stress cardiac magnetic resonance imaging

**DOI:** 10.1186/1532-429X-10-8

**Published:** 2008-01-31

**Authors:** Guenter Pilz, Markus Klos, Eman Ali, Berthold Hoefling, Roland Scheck, Peter Bernhardt

**Affiliations:** 1 Department of Cardiology, Clinic Agatharied, Academic Teaching, Hospital of the University of Munich, Germany; 2 Department of Radiology, Clinic Agatharied, Academic Teaching, Hospital of the University of Munich, Germany; 3 Department of Medicine II, University of Ulm, Germany

## Abstract

Cardiac magnetic resonance imaging (CMR) with adenosine-stress myocardial perfusion is gaining importance for the detection and quantification of coronary artery disease (CAD). However, there is little knowledge about patients with CMR-detected ischemia, but having no relevant stenosis as seen on coronary angiography (CA). The aims of our study were to characterize these patients by CMR and CA and evaluate correlations and potential reasons for the ischemic findings. 73 patients with an indication for CA were first scanned on a 1.5T whole-body CMR-scanner including adenosine-stress first-pass perfusion. The images were analyzed by two independent investigators for myocardial perfusion which was classified as subendocardial ischemia (n = 22), no perfusion deficit (n = 27, control 1), or more than subendocardial ischemia (n = 24, control 2). All patients underwent CA, and a highly significant correlation between the classification of CMR perfusion deficit and the degree of coronary luminal narrowing was found. For quantification of coronary blood flow, corrected Thrombolysis in Myocardial Infarction (TIMI) frame count (TFC) was evaluated for the left anterior descending (LAD), circumflex (LCX) and right coronary artery (RCA). The main result was that corrected TFC in all coronaries was significantly increased in study patients compared to both control 1 and to control 2 patients. Study patients had hypertension or diabetes more often than control 1 patients. In conclusion, patients with CMR detected subendocardial ischemia have prolonged coronary blood flow. In connection with normal resting flow values in CAD, this supports the hypothesis of underlying coronary microvascular impairment. CMR stress perfusion differentiates non-invasively between this entity and relevant CAD.

## Introduction

The assessment of myocardial ischemia is an essential component for the further diagnostic and therapeutic decision making in patients presenting with angina and suspected coronary artery disease (CAD). However, 10–30% of these patients with diagnosed ischemia show no pathological findings in coronary angiograms [1,2]. Most studies suggested coronary microangiopathy to be the cause for angina in this patient collective [3-5].

The use of cardiac magnetic resonance (CMR) including pharmacologically induced stress perfusion as an emerging non-invasive method for the imaging of myocardial ischemia is supported by rapidly growing evidence of its accuracy in predicting relevant coronary stenosis [6-11]. However, knowledge on its proposed capability for also detecting subendocardial perfusion deficit consistent with small vessel disease [12] is limited. As angiographic correlate in these patients, studies have suggested microvascular perfusion deficit [3,5,13]. For the quantification of coronary blood flow, Thrombolysis in Myocardial Infarction (TIMI) frame count (TFC) [14,15] proved to be a simple, reproducible and objective index [5,16-19]. The aims of our study were

1. to correlate CMR detected subendocardial perfusion deficit proposed for coronary small vessel disease with angiographically determined coronary blood flow by corrected TFC

2. and to compare these findings with CAD.

## Methods

### Study population

During a three month period, we prospectively enrolled consecutive patients scheduled for coronary x-ray angiography (CA) who had previously undergone adenosine stress CMR examination and shown subendocardial perfusion deficit. Patients without perfusion deficit and patients with more than subendocardial perfusion deficit in CMR were enrolled in equal proportions to serve as control groups. The exclusion criteria were an internal pacemaker or defibrillator, contraindications for adenosine infusion, or inability to give written informed consent. Written informed consent was obtained from all patients. Patients with a history of myocardial infarction or in whom Late Gadolinium Enhancement (LGE) could be visualized were excluded from the study. All anti anginal medication and caffein containing beverages were stopped at least 24 hours before CMR examination.

### Study protocol

A 12-lead surface ECG was obtained for each patient. All patients were examined clinically and cardiovascular risk factors such as hypertension, diabetes mellitus, hypercholesterolemia, smoking and family disposition for CAD were assessed. In case of claustrophobia mild sedation with midazolame was offered.

### CMR Examination

All CMR studies were performed with a 1.5T magnetic resonance system (Signa Excite^®^, GE Medical Systems, Milwaukee, USA) using an 8-element phased array surface coil (Cardiac coil, GE Medical Systems). Left ventricular (LV) parameters were measured using long-axis (two-chamber and four-chamber-views) functional steady-state free precession (SSFP) sequence images as part of our clinical routine protocol. After infusion of adenosine at a constant rate of 140 μg/kg per minute over three minutes (Spectris MR injector, Medrad, Indianola, USA) first-pass kinetic of a gadolinium-based contrast agent (Omniscan^®^, GE Healthcare Buchler, Germany; 0.1 mmol/kg) was measured in 4 contiguous short axis orientations at every heart beat using a hybrid gradient echo/echo-planar pulse sequence (echo time 1.2 ms, flip angle 25°, slice thickness 8 mm, field of view 32–34 × 24–25.5 cm, matrix 128 × 96) as previously described [7,20]. Echo time was reduced to 1.2 ms for reducing susceptibility artifact as sometimes seen in gradient echo sequences [21]. Ten minutes after stress perfusion a second perfusion study with the same orientation and with the same setting was performed at rest without adenosine infusion. Ten minutes after this second bolus, LGE images were acquired by using an inversion-recovery prepared gated fast-gradient echo-pulse sequence (repetition time 6.7 ms; echo time 3.3 ms; flip 20°; inversion time individually adjusted; slice thickness 8 mm; rectangular field of view 30 to 34 cm; matrix 256 × 160). Again, three long axes, 4–5 short axes as planned in the perfusion study as well as contiguous short axes views using a 3D 20 slice sequence were acquired.

### CMR analysis

**Figure 1:**
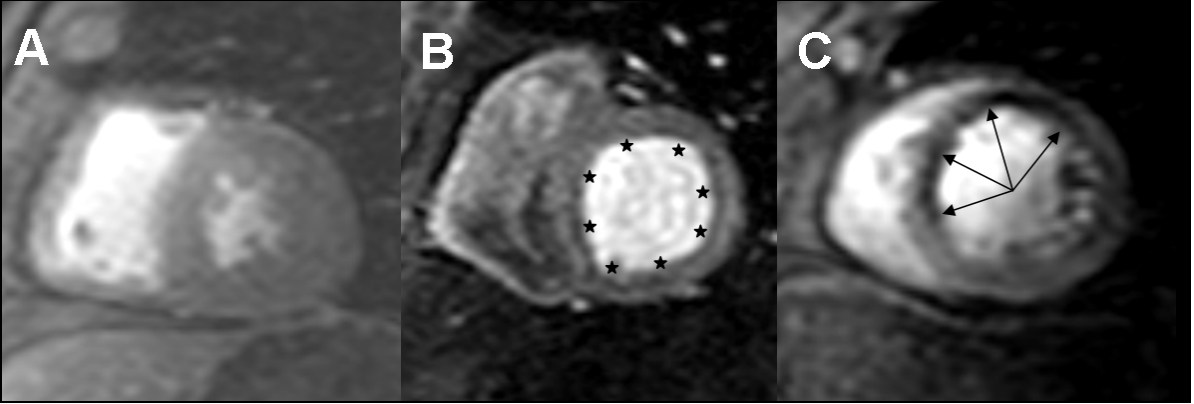
**Adenosine-stress CMR perfusion images**. With (A) no perfusion deficit, (B) diffuse circumferential subendocardial perfusion deficit and (C) perfusion deficit affecting more than subendocardial layers in the LAD perfusion territory.

Two experienced investigators evaluated all CMR studies in consensus. If consensus could not be achieved, a third opinion was included. Image analysis was performed with the standard software provided by the CMR system manufacturer (Advantage Workstation, GE Medical System). Image analysis was performed visually for reducing the rate of false positive results due to rim artifacts as previously reported [22]. Secondly, we compared stress to rest perfusion to reduce the potential rate of artifacts. If a deficit was equally present at stress and rest, if it did not follow the subendocardial border, if ghosting artifacts could be seen or if it "blinked" bright and dark it was not regarded as an evident hypoperfusion, but a potential artifact. Such cases were not included into the study. Segments were classified according to AHA recommendations [23] and evaluated for inducible hypoperfusion during the stress first-pass sequence and classified as "no hypoperfusion", "subendocardial hypoperfusion" [12] or "relevant hypoperfusion" indicative of relevant coronary artery stenosis [6,7] in comparison to rest perfusion images according to following criteria:

- Patients were classified as having small vessel disease, if diffuse subendocardial hypoperfusion [12] (affecting ≤ 1/3 of myocardial wall thickness and myocardial areas supplied at least by two different coronary arteries or circumferential perfusion deficit) and lasting for maximum five heart beats after maximal signal peak intensity in the LV cavity [24]. Patients with a lesser degree of hypoperfusion (such as ≤ 1/3 of myocardial wall thickness in only one territory) were not included.

- Patients with regional perfusion deficit of > 1/3 wall thickness and lasting for more than five heart beats were classified as having CAD with ≥ 70% luminal narrowing [7]. Patients with a lesser degree of regional hypoperfusion (such < 5 beats of stress perfusion defect regardless of affected myocardial wall thickness or ≥ 5 beats but ≤ 1/3 of myocardial wall thickness in one territory) were not included. See figure 1 for examples of our patients groups.

Patients with small vessel disease were analyzed as study patients, patients with no perfusion deficit as "control 1" and patients with perfusion deficit consistent with CAD as "control 2". Classification was performed before CA.

### Coronary angiography

All patients underwent CA within 48 hours after CMR examination. Angiographic case study data were collected and analyzed for affected coronary arteries and degree of luminal reduction.

Afterwards stored angiograms were assessed for corrected TFC for each coronary vessel [14]. TFC analysis was performed by the physician in charge with CA evaluation before CMR data disclosure. The corrected TFC is the number of cine frames required for contrast to first reach standardized distal coronary landmarks [14,15]. In our patients, CA was performed by hand injection of contrast medium via 5 French catheters and filmed at 12.5 frames/s. Since the original TFC is described for a cinefilm speed of 30 frames/sec, an adaptation was performed as previously reported [15]. Therefore, frame counts were multiplied by 2.4 (correction factor). TFC for coronary arteries with normal flow is different in the left anterior descending (LAD) compared to the left circumflex (LCX) and right coronary artery (RCA) because of the larger vessel length. To adapt those differences and to provide comparable results of TIMI frame count for the different vessels, TIMI frame count of the LAD was divided through a correction factor of 1.7 [15].

### Statistical analysis

Data are reported as mean ± standard deviation. Continuous variables between groups were compared by t-test for unpaired observations. Nominal data were compared by Fisher's exact test. Categorial data were compared by Wilcoxon signed rank test for matched pairs. Correlation was assessed by means of correlation coefficient κ and regression coefficient R^2^. In all cases, a p value < 0.05 was considered statistically significant. 95% confidence intervals (CI 95%) are given. Analyses were performed with commercially available statistic software (StatView 5).

## Results

Twenty-two patients out of a total of 265 adenosine stress CMR examinations fulfilled the entry criteria and were enrolled to the study. Mean age was 66.0 ± 12.5 years, 14 (64%) patients were male and 8 (36%) female. 9/22 study patients had previous stress testing (8 bicycle ergometry, 1 dobutamine stress echocardiography), all with pathological findings. The remainder had been referred for stress CMR without previous stress testing. Fifty-one patients formed the control groups. Patients' characteristics are given in table 1.

**Table 1:** Patients' characteristics

	study patients (N = 22)	control 1 (N = 27)	control 2 (N = 24)
Age [years]	66.0 ± 12.5	64.6 ± 12.8	66.1 ± 9.3
Gender [masculine]	14 [64%]	16 [59%]	16 [67%]
Hypertension	15 [68%]*	10 [37%]*	16 [67%]*
Diabetes mellitus^††^	9 [41%]^†^	4 [15%]^†^	11 [46%]^†^
Hypercholesterolemia	10 [45%]	10 [37%]	14 [58%]
Smoking	7 [32%]	11 [41%]	10 [42%]
Family history	7 [32%]	8 [30%]	8 [33%]
Angina			
CCS I	9 [41%]	5 [19%]**	14 [58%]**
CCS II	8 [36%]*	18 [67%]*	7 [29%]*
CCS III	5 [23%]	4 [15%]	3 [13%]

* p < 0.05 study patients vs. control 1 and control 1 vs. control 2

^†^p < 0.05 study patients vs. Control 1 and p < 0.02 control 1 vs. control 2

** p < 0.01 control 1 vs. control 2

^†† ^defined as fasting plasma glucose of > 126 mg/dl or 2-hours postload glucose of > 200 mg/dl or symptoms of diabetes mellitus and random plasma concentration of > 200 mg/dl (ADA criteria).

### CMR

CMR examination was performed in all 73 patients without relevant complications or adverse events. Image quality was sufficient for further analysis in all patients with primary investigators consensus in 71/73 cases and inclusion of a third opinion in two cases. Subendocardial perfusion deficit during stress perfusion in comparison to rest perfusion was found in 22 (30%) patients, who formed the study group. In 27 (37%) patients no perfusion deficit could be observed (control 1), a relevant perfusion deficit was visualized in 24 (33%) patients (control 2). Patient groups did not differ significantly in age or gender (see table 1).

CMR-derived LV parameters in study patients compared to controls were as follows: LV mass (g) 135 ± 36 (control 1: 119 ± 31, p: 0.17; control 2: 129 ± 36, p: 0.65), LV ejection fraction (%) 60.3 ± 8.8 (control 1: 61.7 ± 8.1, p: 0.60; control 2: 60.5 ± 7.2, p: 0.92), LV wall stress (N/m^2^x1000) 43.3 ± 8.8 (control 1: 40.0 ± 7.8, p: 0.25; control 2: 41.3 ± 8.6 g, p: 0.46).

Perfusion deficits in control 2 patients were detected in the LAD perfusion territory in 13 [54%], in the LCX territory in 15 [63%] and in the RCA perfusion territory in 12 [50%] cases, respectively.

### CA data and comparison to CMR

CA was performed in all patients without relevant complications. Coronary one-vessel disease (≥ 70% luminal narrowing) was observed in 15 [21%], two-vessel disease in 9 [12%] and three-vessel disease in 6 [8%]. Mean corrected TFC for the LAD was 21.5 ± 4.6 frames, for the LCX 33.0 ± 7.3 and for the RCA 25.9 ± 4.9.

In our study patients (only subendocardial perfusion deficit on CMR exam) no coronary stenosis ≥ 70% could be shown. In contrast, all control 2 patients (relevant perfusion deficit in CMR) had coronary stenoses as visualized by CA. A highly significant correlation between classification of CMR perfusion deficit and degree of coronary luminal narrowing was found (see table 2).

**Table 2:** Comparison of angiographic results with CMR group classification. Control 2 patients had significantly more often coronary stenosis ≥ 70% compared to study patients (p < 0.0001) and compared to control 1 patients (p < 0.0001).

	0–50% coronary stenosis	51–70% coronary stenosis	≥ 70% coronary stenosis
Study patients (22)	19 (86%)	3 (14%)	0
Control 1 (27)	24 (89%)	3 (11%)	0
Control 2 (24)	0	2 (8%)	22 (92%)

**Figure 2:**
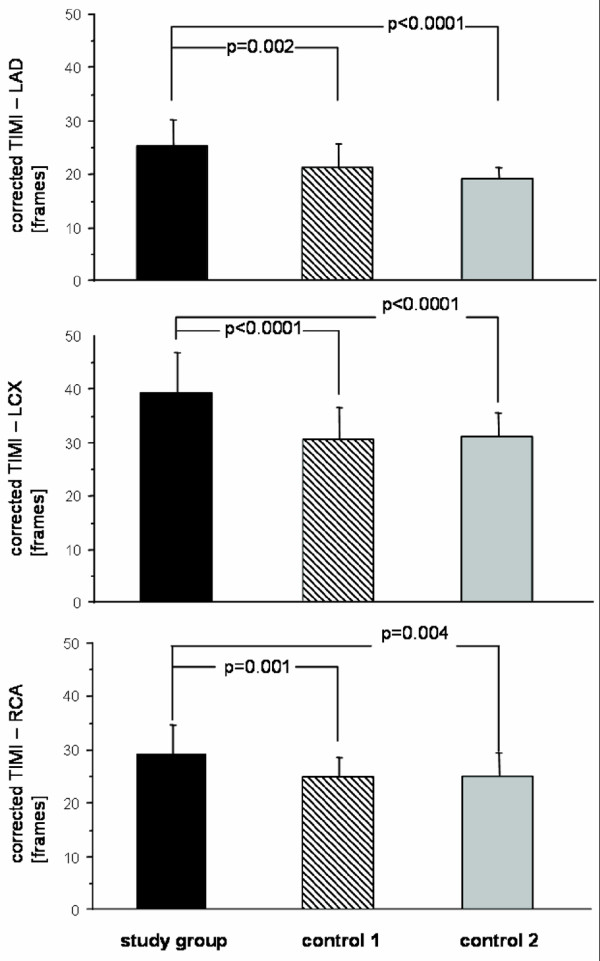
**Comparison of corrected TIMI frame count**. Between study patients and controls for LAD, LCX and RCA. Significantly increased frame count in study patients compared to both control groups.

**Figure 3:**
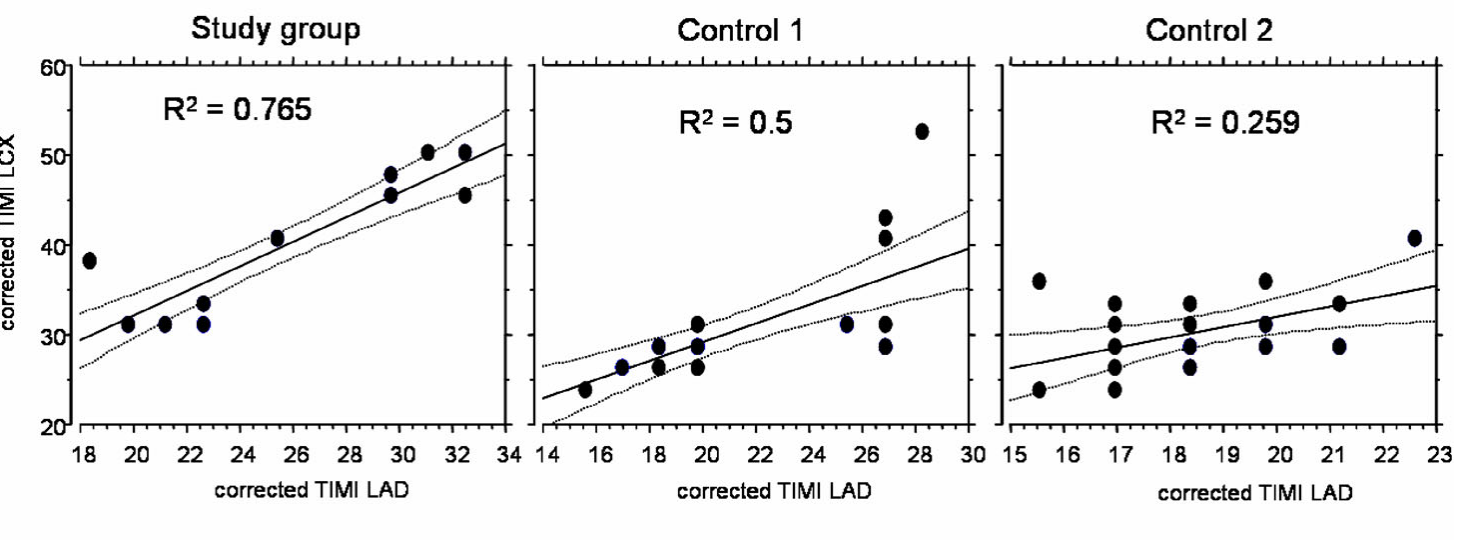
**Regression graphs with 95% confidence interval**. For correlation of LAD and LCX corrected TIMI frame count in our study group and in controls including regression coefficients.

Corrected TFC in all coronary arteries was significantly increased in study patients compared to both controls groups: Study patients vs. control 1 (no perfusion deficit): 25.1 ± 4.9 frames vs. 20.9 ± 4.2 frames in the LAD, p = 0.002; 39.1 ± 7.7 vs. 30.1 ± 6.1 in the LCX, p < 0.0001; 29.1 ± 5.5 vs. 24.4 ± 3.8 in the RCA, p = 0.001) and vs. control 2 (relevant myocardial ischemia): 18.7 ± 2.0 in the LAD, p < 0.0001; 30.7 ± 4.5 in the LCX, p < 0.0001; 24.6 ± 4.5 in the RCA, p = 0.004 (figure 2). Prolonged corrected TFC (values above mean of control 1) were present in all 22 study group patients (per vessel analysis: in 60/66 (91%)), in contrast to control 2 patients (per vessel analysis: in 12/72 (17%)). A good correlation between corrected TFC in LAD and LCX was found in our study patients (κ = 0.87; 95% CI [0.72–0.95]; p < 0.0001). Corrected TFC in control 1 and control 2 patients showed a lower correlation for LAD and LCX (κ = 0.71; 95% CI [0.45–0.86]; p < 0.0001 and κ = 0.50; 95% CI [0.11–0.75]; p = 0.01), respectively (figure 3). No correlation between LAD and RCA or LCX and RCA TIMI frame count was observed, respectively.

Study patients had more often hypertension (15 [68%] versus 10 [37%], p = 0.03) and diabetes (9 [41%] versus 4 [15%], p = 0.04) than control 1 patients. Control 2 patients also had more commonly hypertension (16 [67%], p = 0.03) and diabetes (11 [46%], p = 0.02) than control 1 patients. Patient groups did not differ for hypercholesterolemia or smoking nor did study and control 2 patients differ for the above mentioned cardiovascular risk factors.

## Discussion

Patients presenting with angina pectoris, but having normal coronary arteries with normal ventricular function and without coronary spasm have been described previously [3,4]. Coronary microangiopathy causing increased resistance in prearteriolar coronary vessels, consequently lowering myocardial perfusion and thus leading to impaired coronary flow reserve has been suggested to be the underlying pathophysiology [4,25].

Although having a good long-term prognosis [26], quality of life is significantly impaired in patients with small vessel disease, such as seen in syndrome X, mainly because of persistent angina and decreased exercise tolerance [27]. In addition, diagnostic clarification presently requires invasive angiography.

In the attempt for non-invasive diagnosis, the results of our study confirm that this patients' group can be diagnosed by adenosine-stress CMR examination as first described by Panting et al. [12]. Their study showed patients with syndrome X to have subendocardial diffuse perfusion deficit patterns as seen by adenosine-stress CMR in contrast to patients with a relevant coronary artery stenosis. We used their CMR criteria for our study patient classification with following specification: We added a perfusion deficit lasting five heart beats or less after maximum signal intensity peak in the LV cavity as another inclusion criterion. This is based on the study of Lauerma et al. [24] who showed patients with perfusion deficits lasting more than five heart beats to have relevant CAD. Furthermore, our study extends the protocol of Panting et al. [12] in the following aspects: First, a close time relationship between CMR and angiography (≤ 48 hours in contrast to a mean interval of 18 months [12]); second, use of CMR for patient classification and subsequent angiographic analysis for validation; third, inclusion of two control groups (patients without and patients having relevant perfusion deficit). Most important of all, we focused on the correlation between CMR findings and corrected TFC in these patients.

The main finding of our study was that pure subendocardial perfusion deficit as seen by CMR highly correlates with slowed coronary artery flow as determined by corrected TFC compared to both control patient groups with or without coronary artery stenosis. This finding is consistent with data from a recent study showing patients with small vessel disease to have reduced total coronary blush score [13]. Hence, this correlation between subendocardial ischemia, angina pectoris and reduced coronary artery flow in the absence of coronary artery stenoses strengthens the criteria used for CMR stress perfusion as noninvasive diagnostic imaging modality in the assessment of small vessel disease. Since subendocardial ischemia is a potential source of false positive CMR interpretation of CAD yielding in a lower specificity [11], establishing criteria for detection of small vessel disease may improve specificity and thus, the accuracy of adenosine-stress CMR.

Standard LV parameters such as LV mass, ejection fraction and wall stress were less discriminatory than stress perfusion. In addition, other stress tests yielding findings compatible with myocardial ischemia in the study group were incapable of differentiating from CAD, at least in the subset of patients referred with previous stress tests.

Remarkably, mean resting corrected TFC was normal in our CAD group. TFC measurement during stress was not part of our protocol. Methodologically, it is primarily TFC during stress ("hyperemic TFC") which has been shown to yield decreased values in stenosed arteries, with significant improvement after successful angioplasty with stent placement [16]. In this context, further studies correlating both resting and hyperemic corrected TFC with QCA determined degree of stenosis in CAD are recommended.

Another finding of our study is that in small vessel disease corrected TFC in the LAD correlates very well to that in LCX. This is in concordance with findings of another recent study in patients with microvascular dysfunction and angina pectoris [5].

Furthermore, our results support the concept that systemic hypertension and diabetes mellitus are not only risk factors for CAD in epicardial vessels, but also for small vessel disease. Our data strengthen the hypothesis of microvascular functional impairment in patients with small vessel disease: The slowed coronary artery flow under rest causing detectable pure subendocardial ischemia under stress conditions in the absence of coronary artery stenoses suggests microvascular disease as the cause of anginal symptoms. The causes of microvascular dysfunction are probably multiple in these patients. Structural abnormalities like myocardial medial hypertrophy and/or fibrosis of arteriolar vessels have been described in a small patient cohort [28]. Regarding higher TFC in microvascular disease compared to CAD patients with the same risk factors, we can only hypothesize that microvascular disease and CAD, although sharing diabetes and hypertension as risk factors, form two distinct and not necessarily concurrent disease manifestations.

The following limitations need to be mentioned for our study. First, we did not directly measure coronary flow velocity using a flow wire and have used instead corrected TFC as a surrogate. This approach may be questioned in view of the results of Chug et al [29], who found no significant correlation between coronary flow velocity reserve and corrected TFC in patients undergoing coronary intervention. On the other hand, several studies have shown the validity of such angiographic grading of coronary blood flow by comparing it to reference methods such as Doppler flow wire during baseline [30] and hyperemia [16], flow velocity measured by magnetic resonance [19] or, most recently, by Doppler echo [31]. Flow quantified by TFC is related to the risk of adverse outcomes in acute coronary syndromes [32] or in heart transplant coronary vasculopathy [33], and corrected TFC has been used as endpoint in interventional trials on early recanalization of the infarct-related artery [34-36]. Thus, although use of TFC in our study seems feasible, TFC disaffirmation in the study of Chug et al cannot be disregarded and therefore the use of this non-invasive surrogate parameter is contradictory. In view of this contradiction, studies directly comparing our CMR-based criteria for diagnosis of small vessel disease with invasive coronary flow velocity measurements by flow wire are required for definitive confirmation of our results. In this context, Muehling et al have shown a significant correlation between invasive measurement of coronary flow reserve and noninvasive evaluation by CMR perfusion imaging in heart transplant arteriopathy [37]. In CAD patients, three recent studies have found a good correlation between CMR adenosine stress perfusion results and the invasively measured coronary fractional flow reserve [38-40]. Secondly, we did not perform (semi-)quantitative analysis of CMR perfusion images. Finally, while our study group patients were included consecutively and prospectively, the two control groups were enrolled to achieve equal proportions. Thus, the groups do not reflect the incidence of the three conditions studied. Although the very strict CMR classification criteria should ensure homogenous groups for evaluation, it precludes neither referral nor selection bias. Thus, while syndrome X patients are predominantly females [12], the unexpected high proportion of males in our study cohort was most likely due to referral bias.

In conclusion, subendocardial perfusion deficit as seen by CMR highly correlates to slowed coronary artery flow. Given normal resting flow values in our patients with coronary macroangiopathy, this finding is most likely due to coronary small vessel disease. CMR allows non-invasive detection of these patients.
